# Genomic big data hitting the storage bottleneck

**Published:** 2018-04-19

**Authors:** Louis Papageorgiou, Picasi Eleni, Sofia Raftopoulou, Meropi Mantaiou, Vasileios Megalooikonomou, Dimitrios Vlachakis

**Affiliations:** 1 Laboratory of Genetics, Department of Biotechnology, School of Food, Biotechnology and Development, Agricultural University of Athens, Athens, Greece; 2 Department of Informatics and Telecommunications, National and Kapodistrian University of Athens, Athens, Greece; 3 Sotiria Chest Diseases Hospital, Athens, Greece; 4 Division of Endocrinology and Metabolism, Center of Clinical, Experimental Surgery and Translational Research, Biomedical Research Foundation of the Academy of Athens, Athens, Greece; 5 Computer Engineering and Informatics Department, School of Engineering, University of Patras, Patras, Greece

## Abstract

During the last decades, there is a vast data explosion in bioinformatics. Big data centres are trying to face this data crisis, reaching high storage capacity levels. Although several scientific giants examine how to handle the enormous pile of information in their cupboards, the problem remains unsolved. On a daily basis, there is a massive quantity of permanent loss of extensive information due to infrastructure and storage space problems. The motivation for sequencing has fallen behind. Sometimes, the time that is spent to solve storage space problems is longer than the one dedicated to collect and analyse data. To bring sequencing to the foreground, scientists have to slide over such obstacles and find alternative ways to approach the issue of data volume. Scientific community experiences the data crisis era, where, out of the box solutions may ease the typical research workflow, until technological development meets the needs of Bioinformatics.

## Introduction

Since 1956, but mainly in the last decades, storage space needs have grown spectacularly. The problem is that, as time flows, the storage funding issue has increased more than sequencing. That is a big problem that the modern scientist has to face. Sequencing has become more troubling because this issue makes the whole procedure difficult. The motivation for sequencing and producing new data has started to fall away (De Silva and Ganegoda, 2016).

Such data comes in the form of short sequencing reads, i.e. short character strings (typically having lengths in the range 75–150). Each character represents a nucleotide (which is also called a “base”), and can assume the values of A (adenine), C (cytosine), G (guanine), T (thymine), or N (failure in the base calling process) (Langmead, 2010). The nucleotide string is usually accompanied by a corresponding string of ASCII characters, encoding the “quality” (that is, the error probability of the base calling) of each of the nucleotides. This is a representative case of how a typical sequencing setup works when a resequencing problem is considered. In such a case, a reference (possibly not 100% accurate) for the genome/transcriptome of the organism being sequenced is already known. One has to map the DNA/RNA sequence reads to the reference (*i.e.*, understand where such reads come from in the reference) and find variants present in the genetic code of the specific organisms compared to the reference (Xu *et al.*, 2014).

Depending on the biological application at hand, one might need to perform several tasks on the data, possibly in several steps, with both per-read and global computations required (Libbrecht and Noble, 2015). A typical workflow corresponding to the above use case might be as follows:

- store the reads in compressed searchable form (necessary to avoid excessive storage consumption);
- retrieve (a subset of) the reads based on some criterion, possibly depending on the experiment metadata (for instance, select all the sequencing reads derived from a given tissue subject to a specific biological condition);
- select/process the reads, for example: identify all the reads containing long stretches of low-quality nucleotides, and trim/eliminate them;
- pattern/match the surviving data, read by read, onto a reference genome;
- store the reads and their alignments to the reference genome (that is, the matches found in the genome for each read) in compressed searchable form again.

In the meantime, the Cern data centre has upgraded storage capacity on 200 petabytes, breaking the previous record of 100 petabytes. Information produced every day is one petabyte per second. This leads to lack of space capacity within 3 minutes. Then all this information has to be filtered for any findings which are stored for later use, after three minutes everything is deleted and three minutes is a very short period to trace back all this information (Britton and Lloyd, 2014).

All this data that need to be retrieved and handled is being held up in I/O traffic because of slow processing power (Fan *et al.*, 2014). Even if process power isn’t still satisfying for such needs, there are other ways to slide over this obstacle. Technology and science go on hand by hand, and someone has to think out of the box to solve any occurring problem, without being stuck conventionally. The other suggested path is the information packings. By limiting, not only the data space needed for the information that we already have but also the new information we get, we can go further in a less chaotic and more organised environment by throwing away unnecessary information (repeats) (Fan *et al.*, 2014).

The important thing is to compress information without losing data that is needed. One should keep in mind that not only huge amounts of data will need to be processed each day, but also that some operations might need to be performed incrementally. For instance, the data produced at some point might be used to refine the results obtained from some other data generated previously, implying the reprocessing of a possibly much bigger dataset. For these reasons the development of a robust and extensible high-throughput storage/matching/processing system is necessary. Many other workflows might be envisaged, but most of them share the same skeleton structure, that is storage, retrieval, filtering/processing, and final storage of the results.

Clustering information based on a representative model (in some permissible limits) is an interesting way to approach the problem (Slonim *et al.*, 2005). For instance, when information is recorded in output, the ones that don’t differ from our first recorded ones should not be referred. The differences are the essential information for our search.

To some extent, sequencing data are intrinsically noisy (they depend on chemical reactions which are stochastic in nature) (Alvarez et al., 2015). On the one other hand, high-throughput sequencing techniques have now reached a high degree of reliability, so sequencing errors are relatively rare (Pareek *et al.*, 2011). Also, as mentioned above, sequencing machines provide a quantification of the sequencing error at each nucleotide regarding “qualities”, which can be used to pinpoint problematic nucleotides/regions in the read.

## Storage state of the art

Since several years, under the pressure of increasing volumes of data and due to reduced hardware costs, the view of databases as centralised data access points has become vaguer (Sreenivasaiah and Kim, 2010). Fundamental paradigms of data organisation and storage have been revised to accommodate parallelisation, disreputability and efficiency. The storage mechanics, the querying methods and the analysis and aggregation of the results follow new models and practices. Search has gone beyond the boolean match, being directly linked to efficient indexes allowing approximate matching in domains ranging from string to graph matching (Pienta *et al.*, 2016). The main points of this progress can be summarised as follows.

From row-oriented representation, nowadays the trend is to move to column-oriented representation and database systems (Abadi *et al.*, 2009), which are the evolution of what was called “large statistical databases” in earlier literature (Corwin *et al.*, 2007; Turner *et al.*, 1979). Column-oriented database systems allow high compressibility per column (Abadi *et al.*, 2008), by direct application of existing ratio-optimised compression algorithms (Abadi *et al.*, 2006). Furthermore, several threads are pulling current database practices away from the relational paradigm. Large-scale storage and access may include dynamic control over data layout. Peer-topeer (P2P) overlays are also used in distributed stores, exchanging, e.g., index information to contributing nodes in distributed data warehouses (Doka *et al.*, 2011), where even the queries can be executed in a peerbased fashion spreading the processing load. Another alternative, related to large-scale analysis is the case of Pig Latin (Gates *et al.*, 2009), where a SQL-like syntax is used to provide the data flow requirements for analysis over a map-reduce infrastructure. Other efforts offer partial SQL support, as is the case of Hive (Ashish *et al.*, 2010) and the corresponding query language, named HiveQL.

Recently, parallel databases (*e.g.*, Oracle Exadata, Teradata) allowed high efficiency at the expense of failure recovery and elasticity (Pavlo *et al.*, 2009). Newer approaches and versions of these parallel databases integrate a map-reduce approach into the systems to alleviate these drawbacks, see (Abouzeid *et al.*, 2009) for more information.

The increased availability of low-cost, legacy computers has brought cloud computing settings to the front line. Shared-nothing architectures, implying selfsufficient storage or computation nodes, are applied to storage settings (O’Driscoll *et al.*, 2013). There exist also alternative clouds based on active data storage (Delmerico *et al.*, 2009; Fan *et al.*, 2014) where part of the computational database effort is distributed among the processing units of storage peripherals. Such an example is the case of DataLab (Moretti *et al.*, 2010) where data operations, both read and write, are based on “sets” - essentially named collections of files - distributed across several active storage units (ASUs).

Finally, task-focused storage solutions are devised to face problems in bioinformatics (Hsi-Yang Fritz *et al.*, 2011), social networks (Ruflin *et al.*, 2011) and networkmonitoring and forensics (Giura and Memon, 2010), showing how much data requirements drive the need for research on storage systems. Especially in bioinformatics, there exist approaches that combine compressed storage and indexing under a common approach, based on sequence properties and works on indexed string storage (Arroyuelo and Navarro, 2011; Ferragina and Manzini, 2005). There are cases where the system provides tunable parameters that allow a balance between data reuse and space recovery (Hsi-Yang Fritz *et al.*, 2011), by keeping only the data that may be reused shortly. At this point it must be stressed that there still exist relational databases that are used for high-throughput data storage, an example being the NCBI GEO archive (Barrett *et al.*, 2009) which supports the submission of experimental outputs and provides a set of tools to retrieve, explore and visualise data. However, even in the case of NCBI GEO, the relational nature of the underlying database is used to identify specific datasets and not specific sequences (*i.e.*, instances). Further analysis tools are used to locate sequences and aggregate information from them. In time series and sensor networks, storage can be a severe problem. In the literature, there are methods such as Sparse Indexing (Lillibridge *et al.*, 2009), where sampling and backup streams are used to create indexes that avoid disk bottlenecks and storage limitations.

Beyond the full-text indexing - combined with compressed storage, as explained above - often met in bioinformatics, there are several works on time series indexing and graph indexing. These two types of indexes, together with the string (and, thus, sequence) indexes, provide full artillery of methods that can cope with a great variety of problems and settings. Graph indexing is under massive research, due to its applicability on such cases as chemical compounds, protein interactions, XML documents, and multimedia.

Graph indexes are often based on frequent subgraphs (Yan *et al.*, 2005), or otherwise “semantically” interesting (Jiang *et al.*, 2007). There exist hierarchical graph index methods (Abello and Kotidis, 2003), and hash-based ones. A related recent work (Schafer *et al.*, 2017) relies on “fingerprints” of graphs - derived from hashing on cycles and trees within a graph - for efficient indexing. The method is part of an open source software, named “Scaffold Hunter”, for visual analysis of chemical compound databases.

In the case of time series, to efficiently process and analyse large volumes of data, one must consider operating on summaries (or approximations) of these data series. Several techniques have been proposed in the literature (Anguera *et al.*, 2016), including Discrete Fourier Transform (DFT), Discrete Cosine Transform (DCT), Piecewise Aggregate Approximation (PAA), Discrete Wavelet Transform (DWT), Adaptive Piecewise Constant Approximation (APCA), Approximation (SAX), and others. Recent works (Emil Gydesen *et al.*, 2015) based on the iSAX (Shieh and Keogh, 2009) algorithm have focused on the batch update process of indexing very large collections of time series and have proposed highly efficiency algorithms with optimised disk I/O, managing to index “one billion time series” very efficiently on a single machine. Another system, Cypress (Reeves *et al.*, 2009), applies multi-scale analysis to decompose time series and to obtain sparse representations in various domains, allowing reduced storage requirements. Furthermore, this method can answer many statistical queries without the need to reconstruct the original data.

## Conclusions

The life sciences are becoming a “big data business”. Modern science needs have changed, and lack of storage space has become of great interest among the scientific community. There is an urgent need for computational ability and storage capacity development. In a short period, several scientists are finding themselves unable to extract full value from the large amounts of data becoming available. The revolution that happened in next-generation sequencing, bioinformatics and biotechnology are unprecedented. Sequencing has to come first in priority but, because of technical problems during this process, the time spent to solve space problems is longer than the one dedicated to the part of collecting and analysing data. During this problem, a huge amount of data produced every day is being lost. As we understand, the scientist must overcome some hurdles, from storing and moving data to integrate and analysing it, which will require a substantial cultural shift. Moreover, similar problems will appear in many other fields of life science. As an example, the challenges that neuroscientists have to face in the future will be even greater than those we nowadays deal with the next generation sequencing in genomics. The nervous system and the brain are far more complicated entities than the genome. Today, the whole genome of a species can fit on a CD, but in the future how we will handle the brain which is comparable to the digital content of the world. Therefore, new technological methods more effective and efficient must be found, to serve the needs of scientific search. Solving that “bottleneck” has enormous consequences for human health and the environment.
